# Correction: Facilitating the propagation of spiking activity in feedforward networks by including feedback

**DOI:** 10.1371/journal.pcbi.1013639

**Published:** 2025-11-03

**Authors:** Hedyeh Rezaei, Ad Aertsen, Arvind Kumar, Alireza Valizadeh

An additional affiliation is missing for the third author. Arvind Kumar is also affiliated with the Science for Life Laboratory, Solna, Sweden.
