# Supplementary material for: Facilitating the propagation of spiking activity in feedforward networks by including feedback
Source: PLoS Comput Biol. 2020 Aug 10;16(8):e1008033. doi: 10.1371/journal.pcbi.1008033 (PMC7444537; doi:10.1371/journal.pcbi.1008033)
Supplement: Attachment — Submitted filename: HR_PCOMP_reply_letter.pdf [file pcbi.1008033.s007.pdf]

Dear Dr. Gershman
We apologize for the delay in resubmission. The reviewers raised several interested points and it took us some time adequately address all their concerns.

We would like to thank the reviewers for their constructive feedback which has helped us greatly in improving the presentation of our results. But we would also like to note that we constantly struggled with the dismissive attitude of the second reviewer who at times criticized our approach for the lack of experimental support and then some of his/her comments expressed complete ignorance of very basic facts from electrophysiology.

We are happy to report that we have addressed all their concerns and revised the manuscript according to their suggestions. The changes in the manuscript are marked in blue. In the following we provide a point by point reply to the reviewers' questions and provide pointers in the manuscript where corresponding changes can be seen. All the new text in the manuscript is also marked in blue.

Sincerely
Arvind Kumar on behalf of the co-authors

Reviewer #1: The authors further explore the Communication-through-Resonance mechanism for feed-forward propagation of input pulses through a chain of network modules, by exploring solutions to make transmission faster and more efficient, by being enabled by a smaller number of input pulses. To do so, they modify the original fully feed-forward network architecture, by introducing a "resonance loop" at the initial stage of the chain, which boosts the ignition of the propagation mechanism by facilitating resonance. I personally found the manuscript very interesting and also quite well written with a very pedagogic and natural sequence of presentation of the different results. Also comparisons with the original feed-forward architecture are systematically performed, making transparent the presentation of the effects of the novel ingredient. I am therefore positive about the possibility for this study to be published after revision.

We thank the reviewer for appreciating our work. In the following we explain how we have addressed the reviewer's concerns in our revised version of the manuscript.

I have however some questions/suggestions for discussion/improvement.

MAYOR ISSUES:

1a) A first question is about Figure 3 shows a distribution of the number of oscillatory cycles induced by the presentation of a single pulse at the input stage. It peaks around 10-12 cycles and have a tail reaching over 50 cycles. Now, when looking at empirical distribution of induced gamma bursting event in visual cortex (e.g. the ones by Shapley's lab, cited in the submitted manuscript) one realizes that it is extremely unlikely to observe in vivo oscillatory bursting lasting more than 5-to-6 cycles. The authors describe their system as being in an asynchronous irregular state that can transiently resonate to boost propagation, contrasting it with Communication-through-coherence (CTC) where a "carrier wave" must be present for long times. Now, it seems to me that what the system is doing here is not producing short oscillatory transients on top of an asynchronous state, but actually triggering a collective transition from Asynchronous Irregular to a synchronous regular regime in the sender pair (if not even in the entire network). Indeed, in Figure 2i one sees that a single pulse induces very quickly a long-lasting in-phase (or slightly out-of-phase) oscillation in the first two layers (1 and 2) forming the resonance pair. It is probably then the persistence of this oscillation that allow CTR from layer 2 to the outer layers to occur. It may thus be misleading to say that the mechanism here at play does not require mechanism that does not require "coherent spontaneous oscillations in the sender and receiver networks". Indeed, the propagation from layer 2 to layer 3 and above (maybe) does not require the oscillation, but without an oscillation in the sender pair this propagation would not occur. Indeed, the sender module is in a real strong induced oscillatory mode as an effect of the stimulus. What this induced oscillation is doing in Figure 2i is producing an internally-generated repetition of the pulse (while in the feed-forward case of Figure 2f the pulse is repeated at the level of external input). An oscillation at the sender level is thus needed to generate internally this input pulse repetition. But is this really compatible with empirical observations, where regular, long-lasting oscillatory transients are never observed? Could the system be made working with shorter "ignition oscillatory transients"?

We thank the reviewer to raise this important issue. To address this concern, we first reduced the number of oscillation cycles induced by a single pulse-packet. To achieve this, we set the network operating point further from the transition border to the synchronous regular regime. This was done by incorporating stronger effective inhibition in our network model, by increasing the Poisson input rate to the inhibitory neurons. Only a 1.6% increase in the input to the inhibitory neurons was sufficient to considerably decrease the number of oscillation cycles, as shown in the distribution of the number of oscillation cycles in the tenth layer of the RPN in Figure 1a below.

We have now replaced Figure 3 in the revised manuscript accordingly. Moving the network operating point away from the transition border by increasing the input rate to the inhibitory population did not impair the pulse-packet propagation, as shown in Figure 1b below.

Regarding the 'coherent spontaneous oscillations in the sender': It is correct that stimulation with a single pulse packet evokes coherent persistent oscillations in the first two layers (resonance pair). But from the third layer onwards there were no persistent coherent oscillations. Thus, this is clearly different from CTC in which coherent oscillations must exist before the arrival of the pulse-packet. In our model, there is a simple explanation why oscillations in the first two layers (the resonance pair) are coherent – because of the bidirectional coupling between the layers. In CTC, we are not sure of the mechanisms underlying the coherent oscillations. Therefore, we would like to argue that the mechanism we proposed is different from CTC, even though it may entail coherent oscillations in the resonance pair. We have now added text accordingly in the Discussion Section to clarify this point [See lines 628-680].

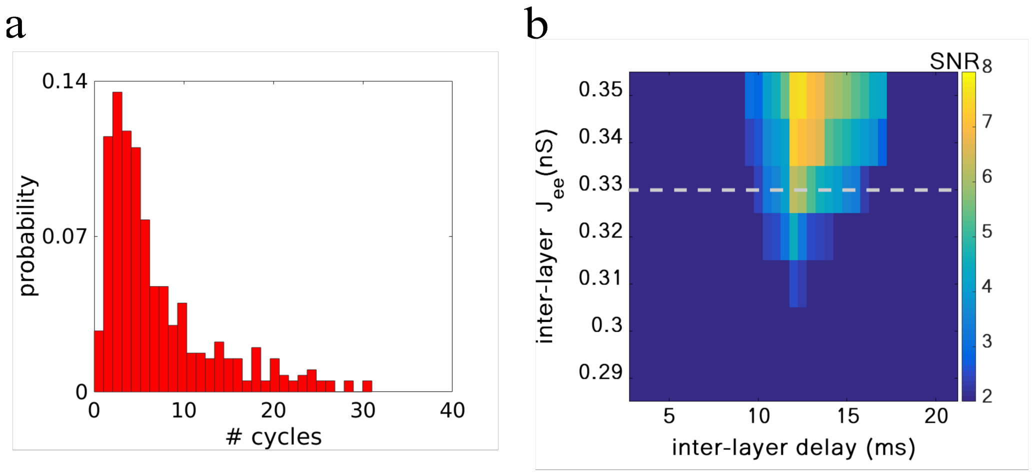

Figure 1: (a) Distribution of the durations of oscillatory activity in the tenth layer of the RPN upon injection of a single pulse packet into the first layer. The RPN, when operating in a successful propagation mode, was able to quench the stimulus-induced oscillations after only few oscillation cycles. The distribution of oscillation durations (shown in units of oscillation cycle) had a median of 5 cycles. These data were collected from 400 trials. (b) SNR of the tenth layer of the RPN as a result of injecting a single PP into the first layer. Increasing the Poisson input to the inhibitory population decreased the number of oscillation cycles, but did not change the propensity of the network to propagate the PP for matching delays.

1b) In general, more developed comparison between this oscillation-induced CTR with models of oscillatory-transient-based CTC could be useful (the burst-mediated CTC of Palmigiano et al. 2017 e.g. relies on much shorter on average oscillatory transients than shown here in Figure 2i, however the efficiency of communication is much smaller).

We indeed see a relationship between the mentioned study and ours. In the Palmigiano et al. 2017 study, the transient oscillation between the two coupled populations occurred simultaneously and showed correlation. Hence, it could be explained by the fact that oscillations in one population supported the induction of oscillations in the other. The difference with our model, however, is that in the model used by Palmigiano et al. oscillations were spontaneous, because the networks were operating presumably closer to the oscillatory mode, and connection delays were smaller. This suggests that the oscillations were not induced by reverberations between two networks as in our model. In our model, the loop delay was equal to the intrinsic network oscillation period and the oscillations were evoked by the pulse packet stimulation. With

the pulse packet stimulation, the resonance pair network was operating in an aperiodic asynchronous activity regime. We have now added text accordingly to clarify this point in the Discussion Section [See lines 649-663].

2a) What if rather than a single pulse, a train of pulses is applied? Before the single pulse can be propagated an entire ignition oscillatory epoch must occur in the sender pair before propagation to outer layers starts. So what if a second pulse input arrived during this ignition oscillatory transient? Would this reset the wave, slowing down propagation or even preventing propagation of the first pulse? Would the two pulses be mixed or information about the presence of two pulses in a sequence would still retained in some way?

First of all, we would like to emphasize that the inclusion of a resonance pair into the network precludes the need of regular pulse train (PT) stimulation (see Fig. 2 and the associated text in the revised manuscript). However, if the input were indeed a periodic pulse train, its propagation would depend on the frequency of the pulse train. To address the reviewer's question, we applied a periodic PT to the RP and studied the interaction between the endogenous (due to the RP) and exogenous (due to the PT) oscillations. The result is shown here in Figure 2 which, together with the associated text, is also included as Supplementary Figure S3a in the revised manuscript. Please see also lines 333-344 in the revised manuscript.

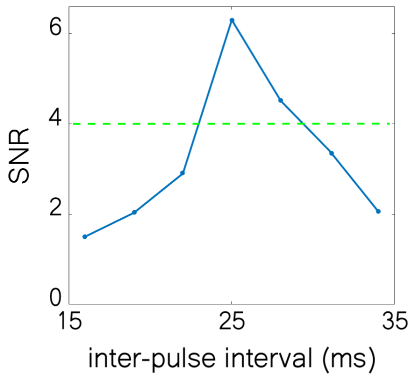

Figure 2: In the presence of both endogenous (RP) oscillations and exogenous periodic pulse train input (PT), the network could propagate the signals with matching inter-pulse intervals. Inter-layer delays were chosen to match the resonance period of the network. The green dashed line represents the lowest amount of SNR for which propagation could be considered successful.

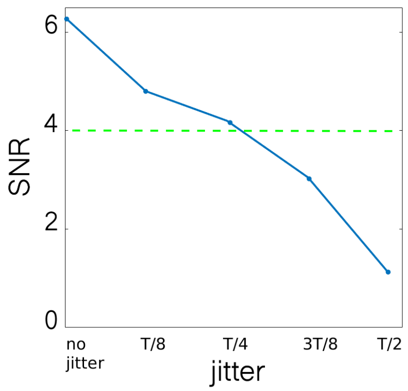

Figure 3: The RPN was stimulated by a non-periodic PT with some jitter in arrival times (shown along the X-axis).While for jitters of up to one fourth of the network's resonance period we observed successful propagation of non-periodic PTs, for larger jitters the SNR in the tenth layer fell dramatically to values below SNR = 4.

2b) A related question. One could think about presenting not one single pulse but a train of pulses. The some questions arise. For instance, could a temporally irregular (non periodic) train of pulses be transmitted?

Before replying to this question, we would like to point out that already in the original network model described in Hahn et al. 2014, propagation of temporally irregular pulse trains was supported by the CTR mechanism (see Fig. 5 in Hahn et al., 2014).

In case of applying an irregular PT to the RPN, we face an interesting question: how aperiodic are the pulses in the PT? To control the amount of irregularity, we jittered the pulses by a small amount (dt), while keeping the mean pulse interval constant. The amount of jitter (dt) was quantified in terms of the network's resonance period (T). We found that as the jitter increased (making the PT more irregular), propagation of the PT suffered. Specifically, we found that signal propagation was possible only when pulses were jittered by an amount smaller than T/4 (Figure 3 above in this document). We included these results and the associated text as Supplementary Figure S3b in the revised manuscript. Please see the lines 333-344 in the revised manuscript.

2c) Or what if the frequency of the exogenous pulse train does not match the natural frequency of the sender module (which gives the frequency of the "endogenous train of pulses" generated in Figure 2i by a single exogenous pulse). Could the network still be able to transfer trains with unnatural frequencies if delays are adjusted differently than to match the intrinsic frequency of the sender module?

As described above (cf. Figure 3 above), the FFN with RP has only a small bandwidth for propagating regular PTs. We have not tested what will happen if the sender is transmitting at unnatural frequencies and delays between sender and receiver are varied to match the unnatural pulse train frequency. The natural frequency of oscillations is a local property of the sender and/or receiver networks and it is determined by the within-network delays and neuron time constants. Because for pulse packet transfer we rely on the build-up of resonance, it is important that both stimulation frequency and the loop delay (sender <--> receiver connection delays) match the natural frequency of the sender and receiver networks. Therefore, unnatural frequencies lying outside the communication bandwidth will not be propagated, even if sender-receiver delays are matched to this unnatural frequency.

2b+c) There is indeed a potential confounding factor in the study. One input pulse produce a train of pulses in the sender module. Then after a certain number of pulses propagation to layer 3 and beyond is started. Does it matter that the pulses are rhythmic, with a frequency matching the natural frequency of the sender module? Or it is just important that multiple pulses cumulate gradually lowering the excitability threshold for layer 3?

As we have shown above, it is not necessary for the pulses to be completely rhythmic with a frequency matching the network resonance frequency. Both the pulse train frequency and regularity can be varied within a certain, relatively small range (Figures 2 and 3 above). A certain number of pulses are needed in the resonance pair to build up the resonance, but if the frequency or timing of the subsequent pulses does not match, the resonance will break down and propagation will fail. We have added new text in the revised manuscript to discuss this issue [See lines 333-344] and added a new Supplementary Figure S3.

3) What if the oscillatory resonance responsible for the "ignition oscillatory transient" was generated inside the input layer 1 itself (exploiting the I-E-I loops within the sender layer, tuning e.g. the first layer to be near Hopf bifurcation) rather than by a connectivity loop involving two layers?

This is a very good question. This scenario was in fact the basis of the Hahn et al. 2014 study. In that case, the network layer was tuned close to its Hopf bifurcation point such that a single input pulse packet could elicit damped oscillations. But these were not strong enough to sustain stable propagation of a single PP. The only way to sustain propagation was to maintain the input until the network built up sufficiently strong resonance. That necessity of build-up essentially slowed

down the communication and, therefore, in the present study we sought to find an alternative, as we already mentioned in the Introduction [120-124] and Discussion Sections of the original manuscript [See lines 530-534].

4a) What if the resonant pair was not exactly at the beginning but was somewhere else in the middle of the chain? Checking this would be important to understand if the enhancement of propagation is really just due to resonance at the input stage, or whether the presence of a resonant pair makes the entire chain "collectively resonant".

To this question, the answer is both simple and straightforward: Anything before the RP will behave as described in Hahn et al. 2014, whereas anything starting from and beyond the RP will behave as described in the present manuscript.

4b) On the same line, what if there were multiple resonant pairs, e.g. feedback everywhere (with some symmetry breaking in couplings, to keep a directionality in the chain)?

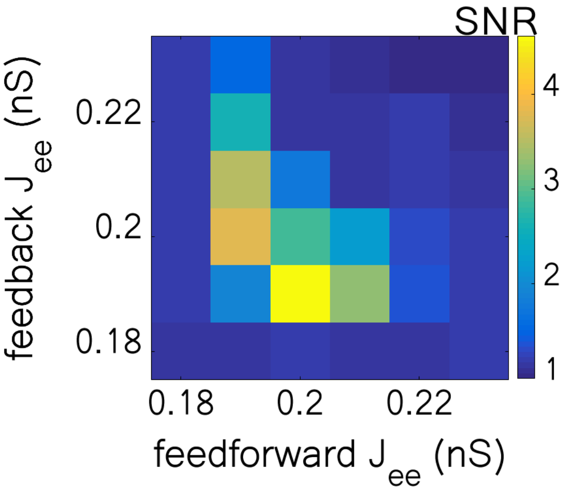

Figure 4: The SNR of the RPN in response to a PP when the resonance feedback connections between 70 excitatory neurons of all layers of the RPN were present with a probability of 0.2. Feedforward and feedback connection strengths were systematically varied. As can be seen, for asymmetric connection strengths for feedback and feedforward synapses the SNR was above the threshold (SNR = 4) and PP propagation was successful.

This is an interesting idea. We tested the effect of such bidirectional connections between all layers in the chain (see Figure 4 below). Having all possible feedback connections led to oscillations in the entire chain, because the inter-layer delays matched the oscillation period. However, in this scenario oscillations depended on the relative strength of the feedforward and feedback connections (Figure 4 below) and delays (not shown). For the results shown in Figure 4 (below), the inter-layer delays were chosen such that they matched the resonance period of the network, and inter-layer connection strengths spanned smaller values when compared to the case of one resonance pair in the network. This could compensate extra excitatory input due to the newly added interlayer connections. The results shown in Figure 4 are very preliminary and a systematic analysis of a chain of bidirectionally connected networks is clearly beyond the scope of this manuscript. We have discussed this issue briefly in the discussion section [see Lines 571-581].

MINOR ISSUES: Is the Fano factor really the best indicator of synchrony? It is an indicator of regularity or irregularity of firing, but at the single neuron level. What about some index explicitly measuring synchronization at the population level?

Yes, the Fano Factor (FF) is one of the simplest and best ways to estimate synchrony in a population. At the population level, i.e. when we sum up the numbers of spikes over the entire population in a small time bin, FF captures the variance of the population activity. This variance is related to synchrony because the population variance is the sum of the individual variances of the neurons and their co-variances (see Kumar et al., J. Neurosci. 2008, Gruen and Rotter 2010). We could use other measures such as average pair-wise correlations but they give essentially the same results, just that the calculation of pair-wise correlations is computationally much more demanding than the FF. [See lines 236-240.]

Figure 7: it may be simpler to add a legend for the three working points, rather than having to read explicitly the caption where the symbols do not visually appear.

We thank the reviewer for this advice and have now replaced Figure 7 with a corresponding new plot, including revised legends.

Figure 8: why so jittery? Are the spectra broadband so that identifying an oscillatory peak is questionable leading to noisy results? Or are the simulations performed too short? Or the fluctuations of peak frequency with the parameters are really so little smooth?

The oscillation frequency appears noisy in the regime where oscillations are weak (high spectral entropy; see panels b and d) and, therefore, it is difficult to determine the peak frequency and, hence, the result is noisy. Once the network enters an oscillatory regime (low spectral entropy), the peak frequency estimate becomes more reliable.

Reviewer #2: General

The contribution of this work is neither theoretical or neuroscientific, is its a computational advancement on the description of a mechanism already proposed by the group that has not been observed experimentally or understood theoretically. Without an attempt to give a mathematical insight to the phenomenon, or to contextualize it such that has biological relevance I don't think its a significant contribution.

We respectfully disagree with the reviewer here. We would like to argue that our work is strongly rooted in observations on neuronal networks in the brain, both in experiments and in theory. First, it is well known that neuronal networks in the brain have a resonance property (e.g. Cardin et al., Nature 2009). Second, there are indeed (more than expected by chance) bidirectional connections in networks in the brain, both at small scale (<1mm) and at meso-scale (i.e. connections between different brain regions (e.g. Felleman and van Essen 1991, Markov et al. 2014a,b, Gămănuţ et al. 2018). Third, connections among neurons are weak and sparse and, therefore, they require some mechanism to successfully propagate spiking activity across networks. Most likely, there are multiple of those at work in the brain (e.g. see Abeles 1991, Kumar et al. 2010).

As for CTR, while we do agree that there is currently no direct evidence for CTR, indirect evidence does support the idea of CTR and the gradual build-up of resonance in the network. For instance, the well-known stimulus-induced gamma oscillations in the visual cortex (Gray and Singer 1989, Gray et al. 1989, Eckhorn et al. 1988) typically have latencies with respect to visual stimulus onset of well over 100 ms (Eckhorn et al. 1991).

Furthermore, our work is based on a sufficiently solid mathematical foundation. We very well understand when and how oscillations and resonance appear in a network of spiking neurons (e.g. Brunel and Wang 2003, Ledoux and Brunel 2011). While we do not provide an analytical solution of the dynamics seen in the resonance pair network, we do show under which conditions resonance occurs and at what frequency, including the bandwidth of the resonance. To this end, we have systematically explored the role of feedback and feedforward connections and the loop delay (Figures 7 and 8 in the revised manuscript, respectively).

Hence, in summary, we consider it highly unfair and incorrect, if not misleading, of the reviewer to argue that “The contribution of this work is neither theoretical or neuroscientific”.

1) The paper doesn’t explain the reader why this advancement from CTR is relevant. Where is this synchronous propagation in the brain? In which animal? In which system? Are you thinking about evoked gamma propagating through the visual hierarchy (somewhat reported in Roberts et al Neuron 2013)?

We have very clearly argued in the Introduction of our manuscript that an advancement of CTR is needed because CTR as described in Hahn et al. 2014 only allows for a very slow mode of communication [see lines 120-124]. It is clear that in many cases such slow communication will not be functionally relevant. In such cases, we need a distinctly more rapid build-up in the synchrony of the spiking responses or of the CTC. Experimental evidence in this respect is inconclusive. Therefore, we need to explore further possibilities to speed up such communication, one of which is presented in this manuscript.

As for where there is propagation of synchrony in the brain? We would argue that this must occur between most brain regions e.g. between V1 and V2 (in fact it does; see Zandvakili and Kohn 2015), or between CA3 and CA1, or between other cortical and subcortical regions. Whenever we have sparse and weak connections between brain regions, we need to resort to such type of communication, including quite different species (see e.g. Hemberger et al. 2019 Neuron; Putney et al. 2019, PNAS).

The existence of oscillations and a relationship between spike times and oscillation phases suggests a role for oscillations in communication of spiking activity from one network to another [e.g. see numerous papers from the lab of Pascal Fries].

We thank the reviewer for pointing to the Roberts et al. 2013 reference, we have now added this reference [See line 676].

2)The study uses biological motivation to justify new choices but the final implementation seems arbitrary (only first two layers are bidirectionally connected). Figure 12 of Markov et all 2011 Cerebral Cortex shows that V1 receives more connections from V4 than V4 does from V1. I understand the idea of a hierarchy but this is a very strong simplification without an attempt to show how robust it is. A possible way forward is to have weaker degrees of bidirectionally when going through the hierarchy and show robustness. What happens if other layers are bidirectional? How weak the connections between distant layers would have to be for the phenomenon to hold?

This was also suggested by the first reviewer: Having bidirectional connections between all layers and applying asymmetry in the forward and backward connections. We have briefly explored the consequences of bidirectional connections between adjacent layers of an FFN (Figure 4 above). Having all possible feedback connections led to oscillations in the entire chain, because the inter-layer delays matched the oscillation period. However, the occurrence and strength of oscillations depended on the relative strength of feedforward and feedback connections (Figure 4 above) and delays (not shown). For the results shown in Figure 4, the inter-layer delay matched the resonance period of the network. The results shown in Figure 4 are very preliminary and a systematical analysis of a chain of bidirectionally connected networks is a very challenging problem (e.g. see Joglekar et al. 2018 Neuron) and clearly beyond the scope of the present manuscript. We have now mentioned this issue in the Discussion Section [See lines 571-581].

At this point, we would also like to point out that the issue of unidirectional and bi-directional connectivity is indeed a rather contentious issue. As the reviewer pointed out, mesoscopic connectivity either measured by DTI (Markov et al. 2014) or by tracer injections (Harris et al. 2019) does indeed suggest that the connectivity is bi-directional among most pairs of voxels in brain imaging. But this does not mean that functionally the networks are bidirectionally connected. This is because of the following reasons: 1. We still do not know whether connections are equally strong in both directions – DTI and tracer injections techniques are not well suited to resolve this issue. 2. Selective stimulation of a given brain region does not seem to evoke reverberating activity as would be expected from bi-directional connections (e.g. see Logothethis et al. 2010, Klink et al. 2017, Nurminen et al. 2018 for electrical stimulation and optogenetic studies). Starting with the description of the visual information processing areas to the latest mesoscopic connectivity of the mouse brain, there is widespread consensus that there is some sort of hierarchical arrangement of brain areas (*Harris et al. 2019 Hierarchical organization of cortical and thalamic connectivity. Nature*). Therefore, we think that it is reasonable at this point to assume only few truly bidirectionally connected pairs of networks in the brain.

As for whether V4 sends more projections to V1 or the other way around: in our modeling framework this information is irrelevant. While we are not aware of experimental data about the transfer of information from V1 to V4, we can certainly say that there is a unidirectional flow of information from V1 to V2 (Zandvakili and Kohn 2015 Neuron, Semedo et al. 2019 Neuron). So even in the visual neocortex, despite all the evidence of bidirectional connectivity, information seems to flow primarily from V1 to V2.

To discuss this issue, we have added a new subsection in the Discussion Section [See lines 553-570]

3) The authors find that if the delays are fine tuned to match the input period then the propagation is more successful. Despite investing several figures in stating this phenomenon there is no attempt to understand this theoretically. Two E-I networks connected can be studied with linear response theory. Is the network at the onset of a hopf bifurcation? These are LIF neurons with alpha functions, a more complicated version of that was studied in Brunel & Wang 2003 and can be studied with the method developed by richardson even in the nonlinear case (Richardson 2008 Biol Cyber) and in the coupled network studied here.

We would like to point out that the main focus of the manuscript is not to study when and how two populations of excitatory/inhibitory neurons generate oscillations when coupled bidirectionally. A systematic mathematical analysis of the phenomenon, as the reviewer suggests, is quite non-trivial and requires a separate study. For the purpose of this study, we have used numerical simulations to determine the required delays (Figure 4 in the manuscript) and connectivity (both connection probability and feedback connection strengths, Figure 8 in the manuscript) to generate oscillations/resonance that can advance CTR, as was the stated goal of our study, formulated explicitly in its Introduction.

4)The layers are quite small, in which is easier to generate oscillations, what happens for bigger network sizes? How is this network balanced? Do you use BE scaling or theory in any way? A purely balanced net is it inhibition stabilized, is this the case? Which is the fundamental ingredient to get resonance? How can it be understood? Is the effect robust to longer distance connections? and to delay heterogeneities?

To obtain asynchronous irregular activity in Erdos-Renyi type random networks of excitatory and inhibitory spiking neurons, we need to ensure that the average inhibition received by a neuron is larger than the average excitation (Brunel 2000). The Fokker-Plank description of the dynamics also provides us with the parameter ranges within which the network will operate close to the Hopf bifurcation. Therefore, we used the Fokker-Planck description (Brunel 2000, Vlachos et al. 2016 to cite a couple of examples) to approximate the parameters which were then fine-tuned. Unfortunately, we do not know the “BE scaling” the reviewer is referring to.

Thus far in our study, we did not consider the issue of heterogeneities. Therefore, in order to show the RPN’s robustness against delay heterogeneities, we considered a normal distribution for all within and inter-layer delays. We studied two illustrative examples with two different standard deviations for delay distributions: sigma=0.1 and 0.2 of its mean. We found that an increase in the width of the delay distribution made propagation more unlikely (Figure 5 below). Unless the reviewer insists, we would prefer not to include these preliminary results in the manuscript as this issue clearly requires more detailed simulations and analyses. This issue has now been discussed in lines 410-419 and we added a new Supplementary Figure S6.

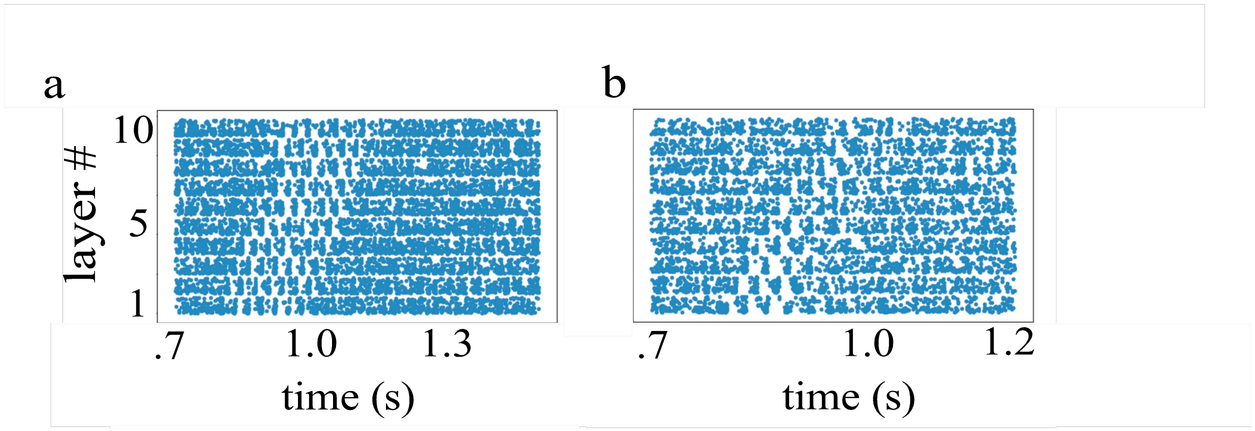

Figure 5: Distributed delays may distort PP propagation, depending on the degree of dispersion of delays. For (a) sigma was 1.25, and for (b) was 2.5 ms. The RPN showed robustness of propagation against the smaller amount of delay dispersion (SNR=4.5), whereas it failed for the case of larger delay dispersion (SNR=2.5).

The fundamental ingredient of the resonance is the mismatch between the amplitude and timing of excitation and inhibition (based on the earlier cited papers from Brunel and colleagues) – we showed this already in Hahn et al. 2014. Here, in the present manuscript, given the construction of the model (bidirectional connections), the EI balance is crucially determined by the feedforward and feedback connections. Therefore, we used numerical simulations to determine suitable delays and feedback connections strengths (Figure 8 in the revised manuscript) to determine the conditions for resonance. We found that for resonance, the loop-delay (sum of feedforward and feedback delays) should match the period of the intrinsic network resonance oscillations (Figures 4 and 5 in the revised manuscript). And we already know from the work of Brunel and colleagues that the oscillation frequency of EI networks depends on the local connectivity and delays.

To quantitatively address the reviewer's concern about larger networks we increased the number of neurons in each layer from 200 excitatory and 50 inhibitory neurons to 2,000 and 500, respectively. As shown in Figure 6 below, matching delays showed higher SNRs and no inconsistency could be seen between the results of large and small networks. Therefore, the network size is not a crucial factor, as long as the feedback and feedforward connection strengths and delays are arranged according to the results shown in Figure 6 below. This result is now included in Supplementary Figure S5 in the revised manuscript and we added new text to discuss this issue [See lines 393-409]

In our model, longer distances imply longer delays. If we increase the inter-layer delays, we will need to reduce the resonance frequency to facilitate spike propagation. Therefore, at long distances, successful propagation will occur only at lower frequencies. In the large network, we have also changed the resonance frequency (as shown in Figure 6b) to 33 Hz by changing the network parameters. In this case, the resonance frequency lies well within the Beta range.

To In summary, we built a clearly larger network with a different resonance frequency, which was distinctly lower than the one reported in the manuscript. As the results summarized in Figure 6 show, the network size does not affect the resonance property of the EI-network, but the network parameters obviously do change its resonance frequency.

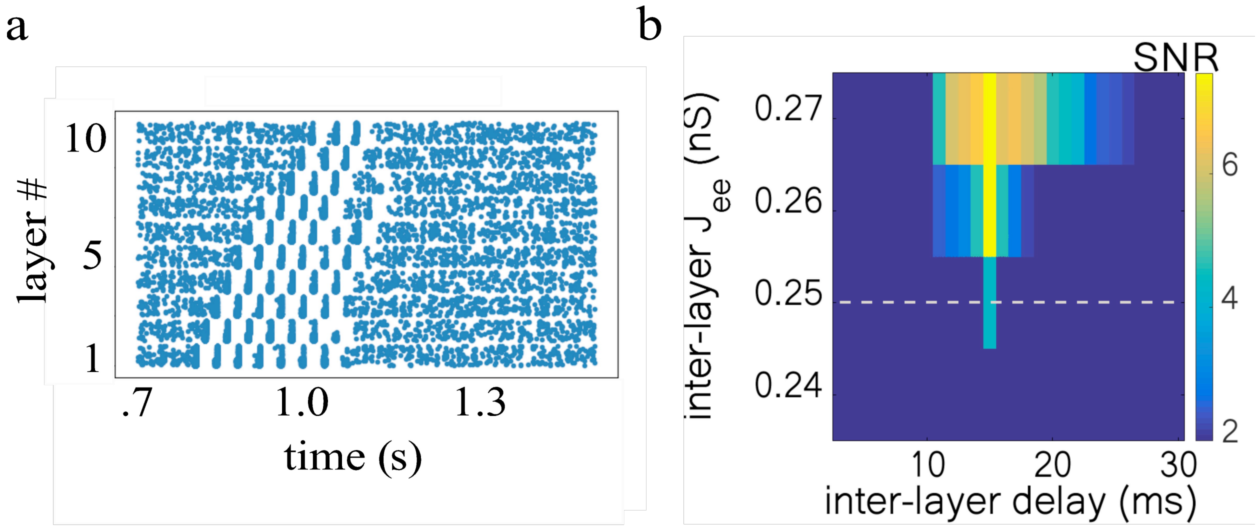

Figure 6: Propagation of a PP across a large RPN consisting of 2,000 excitatory and 500 inhibitory neurons in each layer. (a) The resonance period of the network was 30 ms (this choice also addressed the reviewer's question concerning larger distance connections), therefore, in (b) the SNR peaked at inter-layer delays of around 15 ms, matching the resonance period of the larger network with larger inter-layer delays.

Introduction

1) The introduction seems too close to the original CTR paper, and could improve its degree of argumentation. There is no mention of the plausibility of the CTR mechanism or why would it be interesting to study it. Where does this happen in the brain? What is the model system that you are trying to understand? Is there any evidence for synchronous propagation?

We wish to point out that Reviewer 1 explicitly stated that "*I personally found the manuscript very interesting and also quite well written with a very pedagogic and natural sequence of presentation of the different results. Also comparisons with the original feed-forward architecture are systematically performed, making transparent the presentation of the effects of the novel ingredient.*"

Encouraged by this comment, we decided not to make any changes in the Introduction Section. But, now in the revised version we have added new text in the Discussion Section to discuss the biological plausibility of CTR [See lines 670-680].

2) There is no evidence of unidirectional connectivity, on the contrary the data from Markov and Kennedy indicate that feedback connections are more numerous than feedforward ones, so how do we reconcile these results?

This critique is really not justified. We partly discussed this issue already in our reply to the second point in the General section [Page No. 7 of this document]. But for completeness, we repeat our arguments here once more:

At this point, we would also like to point out that the issue of uni-directional and bi-directional connectivity is indeed a rather contentious issue. As the reviewer pointed out, mesoscopic connectivity either measured by DTI (Markov et al. 2014) or by tracer injections (Harris et al. 2019) does indeed suggest that the connectivity is bi-directional among most pairs of voxels in brain

imaging. But this does not mean that functionally the networks are bi-directionally connected. This is because of the following reasons: 1. We still do not know whether connections are equally strong in both directions – DTI and tracer injections techniques are not well suited to resolve this issue. 2. Selective stimulation of a given brain region does not seem to evoke reverberating activity as would be expected from bi-directional connections (e.g. see Logothethis et al. 2010, Klink et al. 2017, Nurminen et al. 2018 for electrical stimulation and optogenetic studies). Starting with the description of the visual information processing areas to the latest mesoscopic connectivity of the mouse brain, there is widespread consensus that there is some sort of hierarchical arrangement of brain areas (*Harris et al. 2019 Hierarchical organization of cortical and thalamic connectivity. Nature*).

In fact, in most cases, the numbers of connections are clearly smaller in one direction than in the other. In fact, if we threshold these connections, we clearly obtain an asymmetric connectivity matrix, implying stronger uni-directional than bi-directional influences. Perhaps that was the reason that even the latest paper on meso-scale connectivity was entitled: *Hierarchical organization of cortical and thalamic connectivity* [Harris et al. 2019].

Therefore, we think that it is reasonable at this point to assume only few truly bidirectionally connected pairs of networks in the brain. Such connectivity does not need to have equal strengths in both directions. Indeed, in our model we showed that asymmetric connectivity between the two layers of the resonance pair did not affect the build-up of oscillations and the ensuing propagation of PPs (see Figure 8 in the revised manuscript). Hence, we claim that even if there were asymmetric bidirectional connectivity, the resonance effect will be observed.

3) The CTC hypothesis, with all its pitfalls, does stem from experimental findings; whats the experimental evidence for CTR? Also, there are more transmission mechanisms than the ones described that are not even touched upon (Akam, Kullman 2010)

As we described above, there is indirect experimental evidence in support of CTR: [See lines 670-680]

1. Cortical networks do have a resonance property which has been experimentally observed and theoretically studied and understood, as described in detail above, with relevant references to the experimental and theoretical literature.

2. Resonance-induced activity oscillations do take time to build up, as has been observed in experiments and described above (see Eckhorn et al. 1991).

We have added a reference to Akam and Kullman 2010 in the revised manuscript [See line 521].

4) The two limitations to CTC are not fair, one is taken from a paper that says that if CTC is true it would have to be true in small transients, and there is no evidence against that, and the other assertion, that interareal phase locking is not understood theoretically needs a bit more reading (ermentrout kopell, battaglia brunel hansel)

The first limitation of CTC we noted is that oscillations are too short-lived and we should have pointed out that oscillation frequency and phases are also not stable [e.g. see Burns et al. 2011 J. Neuroscience]. But the reviewer is correct in stating that CTC can work even on a single oscillation cycle. As for the synchrony of oscillations, we have already cited recent work from the Bataglia lab which provides a way to synchronize oscillations.

However, we would like to point out that most of the work on phase locking of oscillations is inspired from the work on Kuramoto oscillators. Such understanding cannot automatically be translated to neuronal networks of spiking neurons, with event-driven couplings (as opposed to continuous coupling in Kuramoto systems or rate based models) and when the population shows multiple nested oscillations. In any case, we have now rephrased the text about the limitations of CTC in the revised manuscript [See lines 93-97].

Methods + Results

1)Networks are small, what happens if bigger?

As described above and depicted in Figure 6 above, the basic idea of RPN facilitated propagation is valid, independent of network size.

2)8000 independent poisson? like compound? not clear (l.135)

No, we did not use a compound Poisson process. The sum of stationary Poisson processes is also a Poisson process. We used 8,000 Poisson processes to each spike at 1 spike/sec rate as input. We could have used a single Poisson process with 8,000 spikes/sec as rate and the result would be the same. The main point of this input was to bring the neurons receiving this input to spike at a biologically realistic background rate.

3)reference for spectral entropy

We have added a reference to the paper by Sahasranamam et al. 2016 where spectral entropy was used for the first time to characterize oscillations [See line 255].

4)what is a weak connection? weak compared to threshold? are these numbers close to any experimental data? which one?

By weak connections we mean weak compared to the typical distance to firing threshold of the neurons: i.e. 50-100 weak excitatory inputs are required to drive the membrane potential of neurons to cross threshold. There are numerous experimental evidences for weak synaptic connections. Instead of quoting those various references, we refer the reviewer to a review article by Buzsaki et al. (2014 Nat. Rev. Neurosci.) where the authors show that the synaptic weights are log-normally distributed i.e. most connections are weak. Here, we have chosen the excitatory weight to be 0.3 mV, such that it takes about 50 coincident spikes to elicit an output spike. We have used such values in several of our preceding modeling studies, published in highly esteemed journals (e.g. Diesmann et al., 1999; Mehring et al., 2003; Kumar et al., 2008; Kumar et al., 2010) [See lines 165-169].

are these numbers close to any experimental data?

Yes, they are. An EPSP size of 0.3 mV is close to the mean of the synaptic weight distribution measured in the rat barrel cortex or mouse visual cortex (See Figure 5d in Buzsaki et al. (2014 Nat. Rev. Neurosci.). This reference is now added in the manuscript [See lines 165-169].

As an aside, we wish to note that Reviewer 2, who is posing in his/her review as a solidly based experimental critic of our modeling approach, exposes him/herself here as being totally unaware of elementary electrophysiological facts.

5)tested the idea of connecting the first and second layer in a bidirectional manner (l.228). Where is this idea coming from? As mentioned before there is no evidence for this in the visual hierarchy, are you thinking of another system?

Now we also provide a reasoning why having bidirectional connectivity can help speed up the propagation [See lines 313-319].

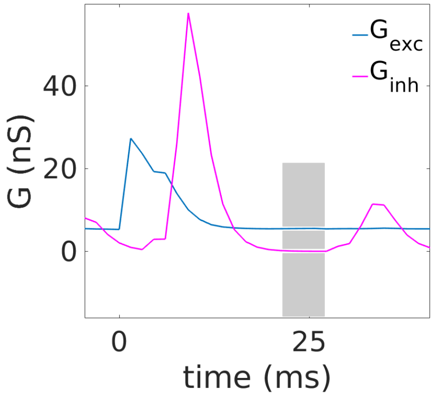

Figure 7: Resonance period of the network was determined as a result of excitatory and inhibitory conductance interplay. A single PP arrived at an isolated layer of an EI network at time 0. The gray window characterizes the most responsive range across which inhibition conductance was in its lowest possible amount. This time window is a direct result of network and synapse parameters.

Assuming bidirectional connections between two networks is not an unusual choice. There are a number of examples of bidirectional connectivity in the brain at different spatial scales. This issue has been discussed earlier when we addressed two previous questions. But to add to that, we were inspired by the fact that at various scales bi-directional connections are known to exist in the brain. E.g. if we look at cortical microcircuits within one layer (<300um), there are more than expected bidirectional connections (e.g. Song et al. 2005, Perin et al. 2011). At a larger anatomical scale, brain regions are bidirectionally connected (e.g. see Coogan and Burkhalter 1988 and Burkhalter 1993 for evidence of feedback connections from V2 to V1). Similarly, hippocampal sub-regions CA3, CA2 have bidirectional connectivity (Ishisuka et al. 1990, Tamamaki et al. 1988). As argued earlier, even though there is an abundance of bidirectional connectivity measured using tracers and DTI, brain stimulation experiments do not show such abundance of bidirectional interactions at spiking activity level (Logothethis et al. 2010, Klink et al. 2017, Nurminen et al. 2018 for electrical stimulation and optogenetic studies). Therefore, it is not a figment of our imagination to consider what function these bidirectional connections might perform.

6)How are these network balanced? balanced networks have strong connections, and you are studying weak connections. Is there any scaling? A balanced network in the classical sense is always inhibition stabilized, is this the case?

We respectfully disagree with the reviewer. From the theoretical literature and from our own published work we very well know that is no such requirement that balanced networks need to have strong connections. The notion of balance can be defined in at least two different ways: (1) The total amount of excitatory and inhibitory input to the receiving neurons is perfectly matched, i.e., the row sum of the connectivity matrix is zero if each row represents inputs received by a neuron (detailed balance). (2) The sum of the entire connectivity matrix is zero (global balance). Moreover, there is the issue of time scales of excitation and inhibition which makes it loose or tight (see Hennequine et al. 2017). But in none of these definitions, the synaptic strength is important. In spiking neuronal networks, just balancing excitation and inhibition is not sufficient to drive the network into an asynchronous-irregular state. To that end, we made our network to operate in the inhibition-dominated regime. Here, as is evident from the synaptic parameters, the

total inhibition was almost 2 times stronger than the excitation (as shown here in Figure 7). That the network was operating in an inhibition-dominated regime is also evident from the statistics of the ongoing activity shown in Supplementary Figure S2 in the revised manuscript.

7)Figure 3. The gamma function there is a bad fit (whats its goodness?). Given that there are simple and really fast simulations, this distribution could be better sampled.

We have deleted the fit and, instead, characterized the distribution of the number of oscillation cycles by its median in the updated Figure 3 in the revised manuscript.

8)Loop transmission delay (l. 249). Why this much is good needs to be understood, finding out something in a simulation is not sufficient. There is not even an intuition of why is this the case.

In Figure 7 of our reply letter, inhibitory and excitatory conductances following a PP injection are illustrated. As can be seen, upon PP arrival first the excitatory conductance increases, followed by a decrease in the inhibitory conductance. The time interval between maximum excitatory conductance and minimum inhibitory conductance provides the most sensitive window for the network by which the resonance period of the network is defined. This value is an outcome of the interplay between neuron and synapse parameters.

9)Fig 8. (b-d) color range doesn't reveal much. Also, again, why is the frequency dependency not smooth? If there is that much variability from pixel to pixel then needs more repetitions to reveal the mean behavior and probably larger networks.

The answer to this question was already given above.

Have all data underlying the figures and results presented in the manuscript been provided?
Large-scale datasets should be made available via a public repository as described in the *PLOS Computational Biology* data availability policy, and numerical data that underlies graphs or summary statistics should be provided in spreadsheet form as supporting information.

Reviewer #1: Yes Reviewer #2: Yes

PLOS authors have the option to publish the peer review history of their article (what does this mean?). If published, this will include your full peer review and any attached files. If you choose "no", your identity will remain anonymous but your review may still be made public.

Do you want your identity to be public for this peer review? For information about this choice, including consent withdrawal, please see our Privacy Policy. Reviewer #1: Yes: Demian Battaglia Reviewer #2: No

References:

- Abeles M. Corticonics: Neural circuits of the cerebral cortex. Cambridge Univ. Press, Cambridge, UK, 1991.
- Brunel N (2000). Dynamics of Sparsely Connected Networks of Excitatory and Inhibitory Spiking Neurons. J Comput Neurosci 8, 183–208.

- Brunel N and Wang X-J (2003). What determines the frequency of fast network oscillations with irregular neural discharges? I. Synaptic dynamics and excitation–inhibition balance. J Neurophysiol 90: 415–430.
- Burkhalter A. Development of feedforward and feedback connections between areas V1 and V2 of human visual cortex (1993). Cerebral Cortex 3(5): 476-487.
- Buzsáki G, Mizuseki K. The log-dynamic brain: how skewed distributions affect network operations (2014). Nat Rev Neurosci 15**,** 264–278.
- Coogan T A, Burkhalter A. Sequential development of connections between striate and extrastriate visual cortical areas in the rat (1988). J of Comparative Neurology 278 (2): 242-252.
- Diesmann M, Gewaltig M, and Aertsen A (1999) Stable propagation of synchronous spiking in cortical neural networks. *Nature* **402,** 529–533.
- Eckhorn et al. (1988) Coherent oscillations: A mechanism of feature linking in the visual cortex? multiple electrode and correlation analysis in the cat. Biol Cybern 60: 121-130.
- Eckhorn R, Schanze T (1991) Possible Neural Mechanisms of Feature Linking in the Visual System: Stimulus-Locked and Stimulus-Induced Synchronizations. In: Self Organiza- tion, Emerging Properties and Learning, Edited by Babloyantz A. New York: Plenum Press; pp. 63-80.
- Felleman D J, and van Essen D C (1991). Distributed hierarchical processing in the primate cerebral cortex. Cereb. Cortex 1, 1–47.
- Gămănuţ et al. (2018). The mouse cortical connectome, characterized by an ultra-dense cortical graph, maintains specificity by distinct connectivity profiles. Neuron, 97(3), 698–715.
- Gray C M, Singer W (1989) Stimulus-specific neuronal oscillations in orientation columns of cat visual cortex. PNAS 86: 1698-1702.
- Gray C M, König P, Engel A K, Singer W (1989) Oscillatory responses in cat visual cortex exhibit inter-columnar synchronization which reflects global stimulus patterns. Nature 338: 334-337.
- Grün S, Rotter S, editors. Analysis of parallel spike trains. Springer Science & Business Media; 2010 Aug 18.
- Hahn G, Bujan A F, Frégnac Y, Aertsen A, Kumar A. (2014) Communication through resonance in spiking neuronal networks. *PLoS Comput Biol*. 10(8): e1003811.
- Harris J A, Mihalas S, Hirokawa K E, et al. (2019). Hierarchical organization of cortical and thalamic connectivity. Nature 575, 195–202.
- Hemberger M, Shein-Idelson M, Pammer L, Laurent G (2019) Reliable sequential activation of neural assemblies by single pyramidal cells in a three-layered cortex. Neuron 104 (2): 353-369.
- Ishizuka N, Weber J, and Amaral D G (1990). Organization of intrahippocampal projections originating from ca3 pyramidal cells in the rat. Journal of Comparative Neurology, 295(4):580–623.
- Joglekar M R, et al. (2018). Inter-areal balanced amplification enhances signal propagation in a large-scale circuit model of the primate cortex. Neuron 98(1): 222-234.
- Klink P C, et al. (2017) Distinct feedforward and feedback effects of microstimulation in visual cortex reveal neural mechanisms of texture segregation. Neuron. 95 (1): 209-220.
- Kumar A, Schrader S, Aertsen A, Rotter S (2008) The high-conductance state of cortical networks. *Neural Computation, 20:1, 1-43.*
- Kumar A, Rotter S, Aertsen A (2010) Spiking activity propagation in neuronal networks: reconciling different perspectives on neural coding. Nature Rev Neurosci 11: 615-627
- Ledoux E, Brunel N (2011) Dynamics of networks of excitatory and inhibitory neurons in response to time-dependent inputs. Frontiers in Comput. Neurosci. 5: 25

- Logothethis N K, et al. (2010) The effects of electrical microstimulation on cortical signal propagation. Nature neuroscience. 13(10):1283.
- Markov N T et al. (2014a). A weighted and directed interareal connectivity matrix for macaque cerebral cortex. Cereb. Cortex 24, 17–36.
- Markov N T et al. (2014b). Anatomy of hierarchy: feedforward and feedback pathways in macaque visual cortex. J. Comp. Neurol. 522, 225–259.
- Mehring C, Hehl U, Kubo M *et al.* (2003) Activity dynamics and propagation of synchronous spiking in locally connected random networks. *Biol. Cybern.* **88,** 395–408.
- Nurminen L et al. (2018) Top-down feedback controls spatial summation and response amplitude in primate visual cortex. Nat Commun 9**,** 2281.
- Perin R, Berger T K, Markram H (2011) A synaptic organizing principle for cortical neuronal groups. PNAS 108 (13): 5419-5424.
- Putney J, Conn R, Sponberg S (2019) Precise spike timing is ubiquitous, consistent, and coordinated across a comprehensive, spike-resolved flight motor program. PNAS 116 (52) 26951-26960.
- Roberts M J, et al. (2013). Robust Gamma coherence between Macaque V1 and V2 by dynamic frequency matching. Neuron 78 (3): 523-536.
- Sahasranamam et al. (2016) Dynamical state of the network determines the efficacy of single neuron properties in shaping the network dynamics. Sci Rep 6**,** 26029.
- Semedo J D, Zandvakili A, Machens C K, Yu B M, Kohn A. (2019) Cortical areas interact through a communication subspace. *Neuron* **102**(1), 249-259.
- Song et al. (2005) Highly nonrandom features of synaptic connectivity in local cortical circuits. PLOS Biology 3(10): e350.
- Tamamaki N, Abe K, and Nojyo Y. (1988). Three-dimensional analysis of the whole axonal arbors originating from single ca2 pyramidal neurons in the rat hippocampus with the aid of a computer graphic technique. Brain research, 452(1):255–272.
- Vlachos I, Deniz T, Aertsen A, Kumar A (2016). Recovery of dynamics and function in spiking neural networks with closed-loop control. *PLoS Comput Biol* 12(2): e1004720.
- Zandvakili A, Kohn A (2015). Coordinated neuronal activity enhances corticocortical communication. Neuron 87 (4): 676-678.
